# Reliability of Macular Ganglion Cell-Inner Plexiform Layer Thickness Measurements Across Scan Protocols in Spectral-Domain Optical Coherence Tomography

**DOI:** 10.3390/jcm15113999

**Published:** 2026-05-22

**Authors:** Nik Krajnc, Martin Bertich, Fabian Föttinger, Stefan Macher, Felix Konstantin Schwarz, Christoph Stapf, Berthold Pemp, Gabriel Bsteh

**Affiliations:** 1 Department of Neurology, Medical University of Vienna, Waehringer Guertel 18–20, 1090 Vienna, Austria; nik.krajnc@meduniwien.ac.at (N.K.);; 2 Comprehensive Center for Clinical Neurosciences & Mental Health, Medical University of Vienna, Waehringer Guertel 18–20, 1090 Vienna, Austria; 3 Department of Ophthalmology and Optometry, Medical University of Vienna, Waehringer Guertel 18–20, 1090 Vienna, Austria

**Keywords:** optical coherence tomography, ganglion cell-inner plexiform layer, measurement reliability, scan acquisition protocols, multiple sclerosis

## Abstract

**Objective**: We aimed to assess whether modified OCT acquisition parameters improve GCIPL measurement reliability. **Methods**: Participants with multiple sclerosis (PwMS) and age- and sex-matched healthy controls (HC) underwent OCT (Spectralis OCT, Heidelberg Engineering, Heidelberg, Germany) at baseline and after two and four weeks. At each visit, five macular scan protocols were acquired: standard (49 lines, high-speed [HS], automated real-time tracking [ART]: 9), high-ART (49 lines, HS, ART: 50), high-lines (97 lines, HS, ART: 9), high-resolution (49 lines, high-resolution [HR], ART: 9), and maximum (97 lines, HR, ART: 50). Reliability was assessed using intraclass correlation coefficients (ICC). Absolute test–retest reproducibility was quantified using the mean absolute difference (MAD). **Results**: Thirty-eight eyes from nine PwMS (mean age 34.1 ± 8.0 years, 44.4% female) and ten HC (31.7 ± 11.1 years, 50.0% female) were included. At baseline, mean GCIPL thickness ranged from 70.8 µm to 71.5 µm across protocols, demonstrating excellent inter-protocol agreement (ICC 0.99; 95% confidence interval [CI]: 0.98–0.99; *p* < 0.001) and only marginally higher values with increased ART. Test–retest reliability was excellent for all protocols, demonstrating marginally lower absolute measurement variability of high-ART and high-lines protocols (MAD 0.26–0.27; 95% CI: 0.21–0.32), while temporal agreement remained excellent and comparable across acquisition settings. Mean acquisition time ranged from 10.6 ± 1.6 s for the standard protocol to 231.9 ± 36.4 s for the maximum protocol. **Conclusions**: All OCT acquisition protocols demonstrated excellent inter-protocol and test–retest reliability for GCIPL measurements. The high-lines protocol provides the most favourable balance between measurement reliability and acquisition time, supporting its potential utility for longitudinal GCIPL monitoring.

## 1. Introduction

Progressive loss of retinal ganglion cells is a central pathophysiological feature of optic neuropathies and is also observed in several neurodegenerative disorders. Among spectral-domain optical coherence tomography (SD-OCT)-derived measures, thinning of the macular ganglion cell-inner plexiform layer (GCIPL) has emerged as a robust structural marker of neuroaxonal loss [1,2,3]. Longitudinal studies have quantified this process directly using SD-OCT. In glaucoma, GCIPL thinning rates are greater in progressing than in stable eyes and may precede measurable visual field loss [1,4]. After acute events such as anterior ischemic optic neuropathy, or optic neuritis, significant GCIPL thinning can be detected within the first few weeks, when peripapillary retinal nerve fiber layer (RNFL) measurements may still be confounded by axonal edema [2,5,6]. In addition, SD-OCT-derived parameters have been investigated as surrogate markers for neurodegeneration in several neurological disorders affecting the central nervous system, including multiple sclerosis (MS), Alzheimer’s disease, and Parkinson’s disease [3,7,8]. Because inner retinal thinning may precede overt visual dysfunction, reliable structural biomarkers capable of detecting subtle longitudinal change are critical for early diagnosis, monitoring, and clinical trial design.

SD-OCT enables high-resolution, cross-sectional visualization of individual retinal layers and provides highly reproducible measurements in eyes without significant retinal pathology [9,10,11,12]. However, the expected annual rate of GCIPL thinning is small: even in glaucoma progressors, rates typically approximate −1 µm per year, and in MS, annualized GCIPL thinning rates frequently remain below 1 µm [1,4,13]. These magnitudes closely approach the limits of test–retest variability, making detection of clinically meaningful change at the individual-patient level technically challenging [14]. As a result, acquisition-related measurement noise may obscure true structural progression and lead to misclassification of stability or worsening in clinical practice and in clinical trials. Current consensus recommendations therefore emphasize strict acquisition consistency and quality control to ensure meaningful interpretation of longitudinal OCT-derived measures [15,16].

Measurement variability in SD-OCT arises from both biological and technical sources, including differences between devices and segmentation algorithms, as well as modifiable acquisition parameters that impact image contrast, segmentation stability and consequently layer thickness mapping [14,16,17,18,19]. Adjustments in scan resolution, the number of averaged image frames, and B-scan density may therefore influence measurement precision and longitudinal sensitivity to change. However, intensified imaging protocols inevitably increase acquisition time and may reduce patient tolerability, thereby introducing a likely trade-off between measurement precision and clinical feasibility. MS, a prototypical inflammatory and neurodegenerative disease, provides a useful model for investigating this issue. Because GCIPL thinning in MS often progresses gradually in the absence of overt clinical deterioration or optic neuritis, it offers an appropriate setting in which to evaluate whether modifications in scan acquisition parameters meaningfully affect longitudinal measurement reliability [20].

Against this background, the present study aimed to determine whether modifications of SD-OCT acquisition parameters materially influence the longitudinal reliability of macular GCIPL thickness measurements and to evaluate their impact on scan duration and clinical feasibility.

## 2. Methods

### 2.1. Patients and Definitions

For this prospective observational study conducted at the Department of Neurology and at the Department of Ophthalmology and Optometry, Medical University of Vienna, we enrolled participants with relapsing multiple sclerosis (PwMS) diagnosed according to the 2017 McDonald criteria [21]. Eligible PwMS were required to be aged ≥18 years and to have been clinically stable (no relapse, no progression) on a stable disease modifying treatment (DMT) for at least six months prior to the baseline OCT examination, with continuous treatment adherence throughout the follow-up period. The patients’ DMT status was classified as following: (1) moderate-efficacy DMT (ME-DMT) defined as PwMS receiving either interferon-beta preparations, glatiramer acetate, dimethyl fumarate, or teriflunomide; or (2) high-efficacy DMT (HE-DMT) defined as PwMS receiving either natalizumab, fingolimod, siponimod, ponesimod, ozanimod, alemtuzumab, cladribine, ocrelizumab, ofatumumab, rituximab or ublituximab. Eyes with ON occurring within six months prior to baseline were excluded. Eyes with a history of ON occurring ≥6 months before baseline, as well as eyes without documented ON but exhibiting inter-eye differences suggestive of prior asymptomatic optic nerve involvement, defined as a ≥4 µm difference in GCIPL thickness, were eligible for inclusion [22]. Both eyes of each participant were included in the analyses unless exclusion criteria were met for the respective eye.

**Figure 1:**
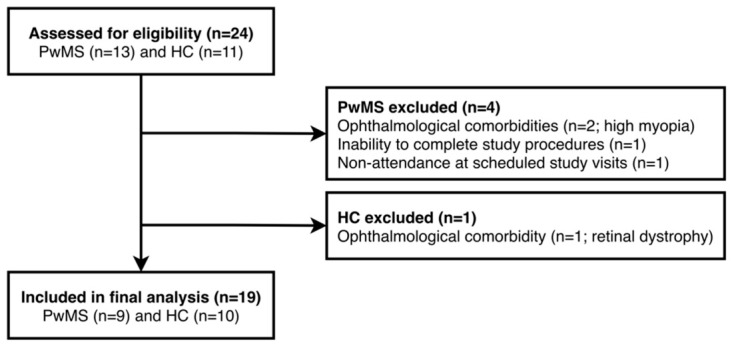
Flow chart of participant enrolment, exclusions, and final study cohort. HC: healthy controls, PwMS: participants with multiple sclerosis.

An age- and sex-matched healthy control cohort (HC) was recruited for comparison. Exclusion criteria for all participants included high myopia (>4 dioptres) and any relevant ocular comorbidity affecting the retina or optic nerve, including glaucomatous, degenerative, vascular, inflammatory, infectious, ischemic, hereditary, metabolic, toxic, or iatrogenic retinal or optic nerve disorders. The selection process based on the inclusion and exclusion criteria is shown in Figure 1.

### 2.2. Optical Coherence Tomography

SD-OCT imaging was performed using the same SD-OCT device (Spectralis OCT, Heidelberg Engineering, Heidelberg, Germany; software Heidelberg eye explorer [HEYEX] version 1.10.4.0) in all participants, in a dark room on both eyes without pupil dilatation. The investigators performing the OCT were blinded to MS clinical parameters. The quantitative OCT study results are reported using the revised Advised Protocol for OCT Study Terminology and Elements (APOSTEL 2.0) recommendations [23]. Additionally, 20° × 20° macular volume scans with horizontal orientation centred on the fovea were acquired repeatedly on each visit.

To assess the impact of acquisition parameters on longitudinal measurement reliability, SD-OCT imaging was performed using five predefined scan protocols, with the standard macular protocol serving as the reference and four modified protocols differing in B-scan density, automated real-time tracking (ART) averaging of image frames, and A-scan rate (lateral resolution). These protocols included:

- Standard protocol: 49 B-scans, high-speed mode (512 A-scans), ART 9.
- High-ART protocol: 49 B-scans, high-speed mode, ART 50.
- High-lines protocol: 97 B-scans, high-speed mode, ART 9.
- High-resolution protocol: 49 B-scans, high-resolution mode (1024 A-scans), ART 9.
- Maximum protocol: 97 B-scans, high-resolution mode, ART 50.

The order of scan protocols was randomized once per participant and then kept fixed across all study visits to ensure consistency within subjects. The same protocol order was applied to both eyes of each participant. This approach balanced between-subject randomization while avoiding within-subject variability related to changing scan sequences across visits. Baseline scans were defined as reference scans, enabling precise alignment of follow-up scans to the identical retinal location through automated eye-tracking. Scan acquisition time was recorded for each protocol and visit.

Image processing was conducted semi-automatically using the built-in proprietary HEYEX software (HRA/Spectralis Viewing Module 6.16.7.0, Heidelberg Engineering). All OCT examinations were reviewed for image quality according to the OSCAR-IB criteria, and only scans that met these criteria were included [15]. The thicknesses of the individual retinal layers were computed in an area of 6 mm centred on the fovea as defined by the Early Treatment Diabetic Retinopathy Study circular measurement grid [24]. Thickness values of the GCL and the IPL were each averaged from the computed values in the quadrants of the 1-to-3 mm and 3-to-6 mm rings of the measurement grid (i.e., excluding the central mm), on an area-weighted basis, and then added together to obtain GCIPL thickness.

### 2.3. Standard Protocol Approvals, Registrations, Patient Consents, and Reporting

The study was approved by the ethics committee of the Medical University of Vienna (ethical approval number: 1478/2024, 19 June 2024). Written informed consent was obtained from all study participants. This study adheres to the reporting guidelines outlined within the ‘Strengthening the Reporting of Observational Studies in Epidemiology’ (STROBE) statement.

### 2.4. Statistics

Statistical analysis was performed using R-Statistical Software (Version 4.4.2). Categorical variables were expressed in absolute frequencies and percentages, continuous parametric variables as mean and standard deviation and continuous non-parametric variables as median with range. Continuous variables were tested for normal distribution using the Shapiro–Wilk normality test.

Descriptive analyses were conducted for GCIPL thickness values obtained with each of the five scan protocols across the entire study cohort at all three time points. Measurement reliability of GCIPL thickness across protocols was assessed by calculating intraindividual coefficients of variation (iCV), intraclass correlation coefficients (ICCs), and mean absolute differences (MADs) [25]. ICCs were calculated with 95% confidence intervals (CIs) using a two-way mixed-effects model with absolute agreement. Comparisons of protocol-specific variability and agreement were performed descriptively by contrasting iCV, ICC, and MAD estimates between each modified protocol and the standard protocol. To assess clinical feasibility, mean acquisition times of the modified protocols were compared with those of the standard protocol using one-way ANOVA. Sensitivity analyses stratified by disease status were performed to assess result robustness with respect to potential disease-related effects.

Statistical significance was defined as a two-sided *p*-value < 0.05, with correction for multiple testing using the Benjamini–Hochberg false discovery rate procedure. Internal validation of the results was conducted using bootstrap resampling.

## 3. Results

In total, 13 PwMS and 11 HC were enrolled. Of these, three participants were subsequently excluded due to ophthalmological abnormalities (high myopia in two PwMS and retinal dystrophy in one HC). One PwMS did not complete the study due to non-attendance to scheduled visits, and one PwMS discontinued the study because of exhaustion caused by fixation instability, which made it impossible to complete the maximum protocol. The final analysis cohort therefore comprised 38 eyes of nine PwMS and ten HC. All PwMS had DMT that was unchanged between visits. Demographic and clinical characteristics of the study cohort are summarized in Table 1. All scans of complete study examinations fulfilled OSCAR-IB criteria and could be included without manual correction of layer segmentation due to annotation errors.

**Figure 2:**
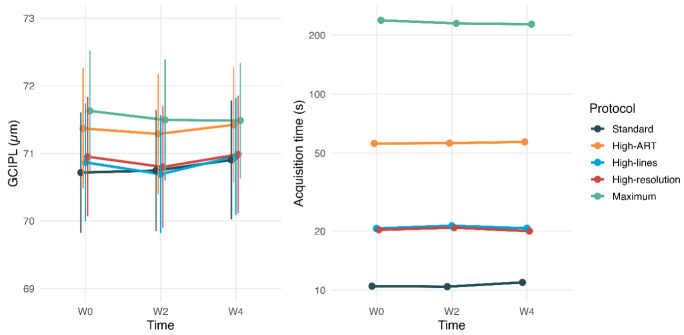
Mean GCIPL thickness (**left**) and acquisition time (**right**) across imaging protocols and time points. Acquisition time is displayed on a logarithmic *y*-axis to accommodate the wide range of values across protocols. ART: automated real-time tracking, GCIPL: ganglion cell-inner plexiform layer, W0: baseline, W2: week 2, W4: week 4.

Mean GCIPL thickness did not differ significantly between study protocols with marginally higher values in both protocols including higher ART settings. However, all four modified protocols exhibited significantly longer acquisition times compared with the standard protocol (Table 2, Figure 2). The pooled mean GCIPL thickness across all protocols and visits was 71.1 ± 5.3 µm, corresponding to an iCV of 0.57%, indicating low variability. Protocol agreement was excellent, with an ICC of 0.99 (95% CI: 0.98–0.99, *p* < 0.001), indicating high reproducibility across protocols. Feasibility of the maximum protocol was limited, as one PwMS discontinued imaging due to fixation-related exhaustion.

**Figure 3:**
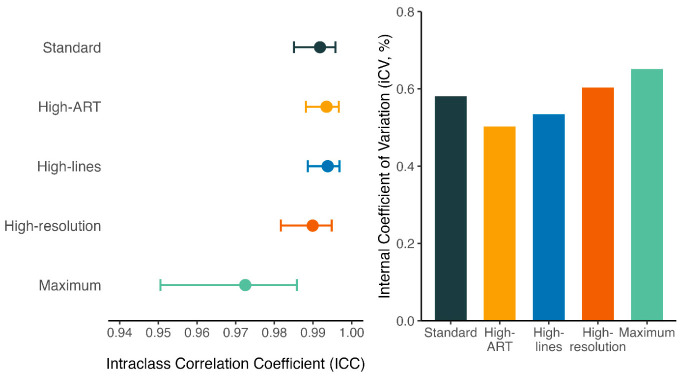
Temporal reproducibility and variability of GCIPL thickness across OCT acquisition protocols. Intraclass correlation coefficients (ICCs) with 95% confidence intervals demonstrating excellent temporal reproducibility across all protocols (**left**). Intraindividual coefficients of variation (iCV) illustrating low relative measurement variability across protocols (**right**). ART: automated real-time tracking.

For the standard protocol, mean GCIPL thickness across time points was 70.8 ± 5.4 µm, with an iCV of 0.58%. Temporal agreement was excellent (ICC 0.99; 95% CI: 0.99–1.00; *p* < 0.001), and the corresponding MAD was 0.30 µm (95% CI: 0.24–0.35). Comparable results were observed for the high-ART, high-lines, and high-resolution protocols, with mean GCIPL thicknesses ranging from 70.8 ± 5.2 to 71.4 ± 5.3 µm, iCVs between 0.50% and 0.60%, excellent temporal agreement (ICC 0.99 for all; 95% CI: 0.98–1.00; *p* < 0.001), and marginally yet not significantly lower MADs for the high-ART (0.26 µm; 95% CI: 0.21–0.32) and high-lines (0.27 µm; 95% CI: 0.23–0.32) compared to the high-resolution protocol (0.32 µm; 95% CI: 0.25–0.40). The maximum protocol showed a mean GCIPL thickness of 71.5 ± 5.3 µm and an iCV of 0.65%. Temporal agreement remained high (ICC 0.97; 95% CI: 0.95–0.99; *p* < 0.001), although slightly lower than for the other protocols, with a MAD of 0.34 µm (95% CI: 0.22–0.53). These protocol-specific patterns of temporal agreement and intraindividual variability are illustrated in Figure 3.

Sensitivity analyses stratified by disease status confirmed that protocol-specific temporal reproducibility was preserved in both PwMS and HC, indicating that disease status did not materially influence the stability of GCIPL thickness measurements. Bootstrap resampling demonstrated high stability of the estimated parameters, with minimal variation across resampled datasets.

## 4. Discussion

In this prospective protocol comparison study, we investigated how variations in SD-OCT acquisition parameters influence the reliability and clinical feasibility of GCIPL thickness measurements over short-term follow-up. Across the standard protocol and four modified macular scan protocols differing in B-scan density, ART averaging, and lateral resolution, GCIPL thickness measurements were highly consistent, with excellent inter- and intra-protocol agreement. Unlike prior work focusing on inter-device or inter-site variability under fixed acquisition settings, we systematically examined the influence of acquisition parameters within subjects [26,27,28]. By varying acquisition settings within the same eyes, visits, device, and software environment, our study isolates acquisition-related technical variance and enables a more granular assessment of protocol-specific effects on longitudinal reliability of SD-OCT from the Spectralis OCT platform.

At the intra-protocol level, all five protocols demonstrated excellent temporal agreement across visits, with high ICCs and intraindividual coefficients of variation well below accepted thresholds [16,27,28]. Importantly, temporal reproducibility was preserved in both PwMS and HC, indicating that disease status did not introduce additional variability under the applied acquisition conditions. This finding supports the interpretation that the observed reproducibility primarily reflects technical robustness of GCIPL measurement rather than disease-specific effects. Such robustness is particularly relevant for longitudinal applications in disorders characterized by gradual retinal neuroaxonal loss, including glaucoma, other chronic optic neuropathies, and neurodegenerative diseases [1,29,30].

Despite the excellent overall agreement, small but systematic differences in absolute GCIPL thickness were observed between certain acquisition settings [31]. Protocols employing extensive ART averaging tended to yield slightly higher GCIPL thickness values compared with the standard protocol, which can be explained by an increase in contrast achieved by averaging a larger number of image frames [14]. Although modest in magnitude and negligible in cross-sectional comparisons, such offsets may become relevant in longitudinal assessments if acquisition protocols are changed between visits, particularly when considered against known biologic sources of GCIPL variability, including age-, sex- and anatomic variability observed in healthy population [12]. In this context, switching between protocols with different ART settings could result in an apparent GCIPL thickness change approaching 1 µm, a magnitude that overlaps with threshold previously used to define neuroaxonal loss and disease progression in longitudinal studies [32,33]. Consequently, even subtle protocol-dependent offsets may confound the interpretation of minor longitudinal GCIPL changes at the individual patient level, underscoring the importance of strict protocol standardization in OCT-based monitoring of neurodegeneration in the optic nerve.

While reliability metrics were uniformly high across protocols, acquisition time differed substantially and emerged as a key discriminating factor. Scan duration increased markedly with enhanced ART averaging and high-resolution acquisition settings, with the maximum protocol requiring nearly four minutes per volume scan—approximately 22-fold longer than the standard protocol. Prolonged acquisition times may limit feasibility in routine clinical practice, increase patient burden, and heighten susceptibility to motion-related artifacts, particularly in populations with reduced fixation stability or limited examination tolerance. Within this context, the high-lines protocol appears to offer the most favourable balance between measurement reliability and practical applicability, providing excellent reproducibility with acquisition times approximately twice that of the standard protocol while remaining substantially shorter than protocols employing high-ART and/or high-resolution settings.

A key strength of our analysis is the combined evaluation of relative reliability and absolute measurement error. Mean absolute GCIPL thickness differences between consecutive visits were uniformly small across all acquisition protocols, with MAD values of approximately 0.3 µm. This degree of variability is substantially lower than reported annual GCIPL thinning rates in glaucoma and neurodegenerative diseases, supporting the feasibility of threshold-based longitudinal interpretation under standardized acquisition conditions [1,32,33]. Consequently, the magnitude of measurement noise observed in this study is unlikely to obscure clinically meaningful structural progression when acquisition protocols are consistently applied.

Although follow-up was limited to four weeks, the observed short-term stability has important implications for longer-term GCIPL monitoring. Stable short-term reproducibility is a prerequisite for interpreting long-term structural change, particularly when the expected annual thinning is small relative to measurement noise. In glaucoma and other chronic optic neuropathies, where progression rates may be subtle and therapeutic decisions increasingly rely on structural progression detection, minimizing acquisition-related variability is essential to avoid false-positive or false-negative progression classification. The low absolute measurement error and high temporal reproducibility observed across protocols suggest that GCIPL thickness provides a robust foundation for longitudinal analyses, provided acquisition protocols and quality control procedures are rigorously standardized. This interpretation is consistent with current consensus recommendations, which emphasize that longitudinal interpretability of OCT-derived retinal measures depends primarily on acquisition consistency, stringent quality control, and transparent reporting rather than maximal technical complexity [23,34].

Several limitations should be acknowledged. First, the modest sample size and short follow-up duration, reflecting the intensive imaging protocol and repeated measures design, limit direct conclusions regarding long-term monitoring and broader generalizability of the findings, although bootstrap resampling supported the stability of our estimates. In particular, the limited number of eyes with prior ON reduced the statistical power of subgroup analyses and may have limited the detection of subtle subgroup-specific effects. Second, inclusion of both eyes per participant may have resulted in partial non-independence of observations. Third, generalizability may be constrained by the use of a single SD-OCT platform operated under standardized conditions; other SD-OCT platforms may allow fewer or different modifications of scan parameters, which could affect the applicability of these findings across platforms and manufacturer-specific acquisition protocols. Finally, the fixed acquisition order may have introduced fatigue-related effects, particularly during more demanding scan protocols.

In summary, GCIPL thickness measurements proved highly robust to variations in OCT acquisition parameters, demonstrating excellent intra-protocol reproducibility with minimal absolute measurement error. While protocol intensification yields only modest improvements in precision, increasing B-scan density provides a favourable balance between reliability and clinical feasibility. These findings underscore that rigorous acquisition standardization, rather than maximal technical complexity, is the critical determinant of reliable longitudinal GCIPL assessment.

## Figures and Tables

**Table 1:** Demographics and clinical characteristics of the study cohort.

	All (*n* = 19)	PwMS (*n* = 9)	HC (*n* = 10)	*p*-Value
Female ^a^	9 (47.4)	4 (44.4)	5 (50.0)	>0.999
Age (years) ^b^	32.8 (9.6)	34.1 (8.0)	31.7 (11.1)	0.592
Disease duration (years) ^c^	NA	2 (0–6)	NA	NA
EDSS ^c^	NA	0 (0–6)	NA	NA
ME-DMT ^a^	NA	1 (11.1)	NA	NA
HE-DMT ^a^	NA	8 (88.9)	NA	NA
Visual acuity (logMAR) ^c^	0.0 (−0.1, 0.1)	−0.08 (−0.1, 0.0)	0.0 (−0.1, 0.1)	0.072
Spherical equivalent (dpt) ^c^	−1.5 (−4.00, 0.0)	−0.75 (−4.00, 0.0)	−1.75 (−4.00, 0.0)	0.304
History of unilateral ON ^a^	NA	2 (22.2)	NA	NA
GCL thickness (µm) ^b^	38.9 (3.2)	38.2 (3.9)	39.6 (2.2)	0.198
IPL thickness (µm) ^b^	31.8 (2.3)	31.3 (2.8)	32.3 (1.8)	0.254
GCIPL thickness (µm) ^b^	70.7 (5.4)	69.5 (6.6)	71.8 (4.0)	0.216

EDSS: Expanded Disability Status Scale, HC: healthy controls, HE-DMT: high-efficacy disease modifying therapy, ME-DMT: moderate-efficacy disease modifying therapy, NA: non-applicable, ON: optic neuritis, PwMS: participants with multiple sclerosis. ^a^ Number (percentage), ^b^ Mean (standard deviation), ^c^ Median (range).

**Table 2:** Mean GCIPL thickness (µm) and scan acquisition time (s) across OCT acquisition protocols and study visits. Values in the “All” row represent the mean across all three study visits.

	Standard		High-ART		High-Lines		High-Resolution		Maximum	
	GCIPL (µm)	Time (s)	GCIPL (µm)	Time (s)	GCIPL (µm)	Time (s)	GCIPL (µm)	Time (s)	GCIPL (µm)	Time (s)
**Baseline**	70.7 (5.4)	10.5 (1.2)	71.4 (5.4)	56.1 (6.1) *	70.9 (5.3)	20.6 (2.7) *	70.9 (5.4)	20.3 (2.0) *	71.6 (5.5)	237.9 (37.7) *
**Week 2**	70.7 (5.5)	10.4 (1.4)	71.3 (5.4)	56.2 (10.0) *	70.7 (5.3)	21.3 (3.8) *	70.8 (5.5)	20.8 (2.6) *	71.5 (5.5)	229.6 (37.2) *
**Week 4**	70.9 (5.4)	10.9 (2.1)	71.4 (5.2)	57.1 (12.8) *	71.0 (5.3)	20.7 (2.8) *	71.0 (5.3)	20.0 (3.0) *	71.5 (5.2)	227.4 (34.1) *
**All**	70.8 (5.4)	10.6 (1.6)	71.4 (5.3)	56.4 (9.8) *	70.8 (5.2)	20.9 (3.1) *	70.9 (5.3)	20.4 (2.5) *	71.5 (5.3)	231.9 (36.4) *
**MAD**	0.30 (0.24–0.35)	NA	0.26 (0.21–0.32)	NA	0.27 (0.23–0.32)	NA	0.32 (0.25–0.40)	NA	0.34 (0.22–0.53)	NA

ART: automated real-time tracking, GCIPL: ganglion cell-inner plexiform layer, MAD: mean absolute difference, NA: non-applicable, OCT: optical coherence tomography. * *p* < 0.001 compared with the standard protocol (one-way ANOVA).

## Data Availability

Data supporting the findings of this study are available from the corresponding author upon reasonable request by a qualified researcher and upon approval by the ethics committee and the data-clearing committee of the Medical University of Vienna.
